# Effect of Autoclaving on the Physicochemical Properties and Biological Activity of Aluminum Oxyhydroxide Used as an Adjuvant in Vaccines

**DOI:** 10.3390/molecules28020584

**Published:** 2023-01-06

**Authors:** Mohamed Skiba, Sofiane Fatmi, Nicolas Milon, Frédéric Bounoure, Malika Lahiani-Skiba

**Affiliations:** 1 UNIROUEN, DC2N INSERM U1239-Galenic Pharmaceutical Team, UFR of Health, Normandy University, 22 Bd Gambetta, 76000 Rouen, France; 2 Laboratory of Marine Ecosystems and Aquaculture, Department of Biological Sciences & Environment, Faculty of Nature and Life Sciences, Abderrahmane-Mira-University, Route de TarguaOuzemmour, Bejaia 06000, Algeria

**Keywords:** aluminum oxyhydroxide, adjuvant in vaccines, biopersistent, autoclaving, amorphous form, physicochemical properties and protein adsorption capacity

## Abstract

The long-term biodistribution of non-biodegradable microstructures or nanostructures used in vaccinations is widely unknown. This is the case for aluminum oxyhydroxide, the most widely used vaccine adjuvant, which is a nanocrystalline compound that spontaneously forms nanoprecipitates. Although generally well-tolerated, aluminum oxyhydroxide is detected in macrophages a long time after vaccination in individuals predisposed to the development of systemic and neurological aspects of the autoimmune (inflammatory) syndrome induced by modified adjuvant. In the present study, we established that the terminal sterilization of aluminum oxyhydroxide by autoclaving in final container vials produced measurable changes in its physicochemical properties. Moreover, we found that these changes included (1) a decreasing in the pH of aluminum oxyhydroxide solutions, (2) a reduction in the adsorption capacity of bovine serum albumin, (3) a shift in the angle of X-ray diffraction, (4) a reduction in the lattice spacing, causing the crystallization and biopersistence of modified aluminum oxyhydroxide in the macrophage, as well as in muscle and the brain.

## 1. Introduction

For decades, aluminum oxyhydroxide (alum), a nanocrystalline compound, has been the most commonly used adjuvant in vaccines. Although alum is generally well-tolerated, it is sometimes reported to cause disabling health problems in people with poorly defined predisposing factors [1,2,3,4,5,6]. The clinical signs attributed to alum are paradigmatic of adjuvant-induced autoimmune and inflammatory syndrome. This syndrome is also observed in patients exposed to silicone gel [4]. The most commonly described symptoms are delayed onset of diffuse myalgias [1], chronic fatigue [5], and stereotyped cognitive impairment [6]. The presence of macrophages loaded with alum is usually detected at sites of previous injections up to 12 years later, causing a specific granuloma known as macrophagic myofascitis [1]. Although biopersistence of adjuvants is undesirable, its exact significance is still debated because the biodistribution of non-biodegradable nanoparticles following injection into muscle is currently unknown. 

It seems important to achieve a fair balance between the efficacy of the alum adjuvant and its potential toxicity [7]. The efficacy and potential toxicity of alum are influenced by whether the modified nanostructure remains localized at injection sites or whether it disperses and accumulates in distant organs and tissues. A study based on IM administration of the Al26 isotope in rabbits showed a low elimination of Al26 in urine (6%) after 28 days. An unknown form of Al26 was also detected in the lymph nodes, spleen, liver, and brain [8]. Aluminum oxyhydroxide is composed of precipitates of submicron-sized nanoparticles. Initially, it was believed that these precipitates remained extracellular until complete dissolution in the interstitial fluid [8]. However, it has been demonstrated that antigen-presenting cells are able to phagocyte alum nanoparticles [9] and, in so doing, to become long-lived cells [10], preventing alum dissolution [4,11,12]. Moreover, this initiates an inflammatory reaction. Inflammatory monocytes are attracted to the muscle by warning signals via a monocyte chemoattractant protein (MCP-1). This stimulation leads to differentiation to macrophages and monocyte-derived dendritic cells before migrating to the draining lymph nodes [13,14]. 

The assurance of sterility is extremely important in the production of parenteral products. The highest level of sterility assurance is obtained by terminal sterilization, which is the exposure of the final product container to steam sterilization or to sterilizing radiation (β,γ). For products that cannot withstand terminal sterilization, which is the case with biologicals and many other products, aseptic processing combined with filter sterilization is used (0.22 μm). The use of aseptic processing provides a lower level of sterility assurance. For this reason, regulatory authorities require terminal sterilization unless the product can be shown to be adversely affected by the sterilization process [15]. The FDA indicates that an evaluation of steam sterilization is required to determine whether a product is terminally sterilizable in the final product, and a written justification is required to demonstrate why a product should not be terminally sterilized. Therefore, terminal sterilization is often used in the manufacture of vaccines [15].

Previous studies showed that the properties of aluminum oxyhydroxide in suspension of aluminum oxyhydroxide in saline solution are changed by progressive heat treatment [16,17,18]. Aluminum oxyhydroxide adjuvant is a crystalline material. Exposure to steam sterilizing conditions causes bonds to form between adjacent Al(OH) _3_ groups and protons of the medium. These changes are manifested as changes in the X-ray diffraction pattern, acidification of the medium, and decreases in the adsorption capacity of proteins [19,20]. Changes in particle size distribution have also been reported by Nail et al. [16].

In this study, we therefore investigated the effect of an additional terminal sterilization on the physicochemical properties of aluminum oxyhydroxide. Aluminum oxyhydroxide products were examined in the final container with and without an additional terminal sterilization process and compared using different analytical methods. 

## 2. Results and Discussion

### 2.1. The Zeta Potential of Aluminum Oxyhydroxide 

Table 1 shows the zeta potential data from aluminum oxyhydroxide samples with and without terminal resterilization. The zeta potential of these aluminum oxyhydroxide samples was approximately 40 mV, which indicates that the net surface charge of aluminum oxyhydroxide was not affected by the additional sterilization treatment.

### 2.2. The pH Measurement of Aluminum Oxyhydroxide Solution

In the pH study, a consistent and reproducible intra- and interday trend of a decreasing of 0.2–0.3 pH units was measured for aluminum oxyhydroxide solutions with additional terminal sterilization (Table 2). The average pH of aluminum oxyhydroxide solutions without a resterilization process was 6.1, and with an additional resterilization process, the average pH was around 5.8–5.9. A low pH indicates that the deprotonation dehydration of aluminum oxyhydroxide occurred as a result of the additional terminal resterilization process, and consequently, the structure of aluminum oxyhydroxide may have been changed [2] ), which was confirmed later by X-ray diffraction analysis.

### 2.3. The X-Ray Diffraction Analysis of Aluminum Oxyhydroxide 

**Figure 1:**
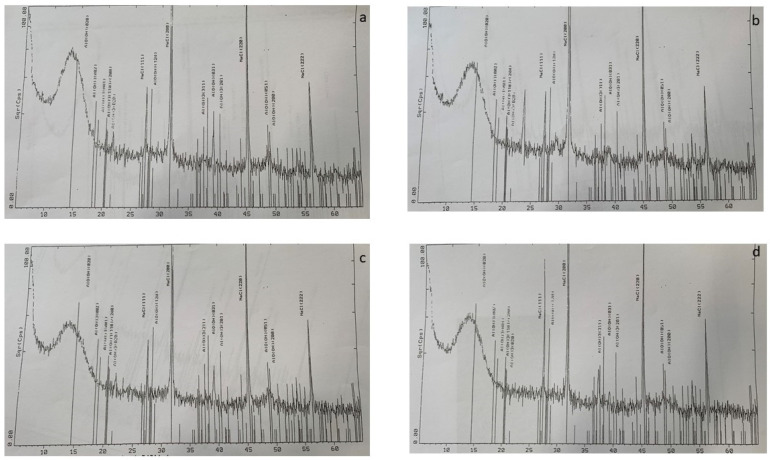
X-ray diffraction analysis of aluminum oxyhydroxide (2θ scale) with (**a**) and without resterilization treatment (**b**–**d**) for 30, 60, and 120 min, respectively. 5-0628: NaCl Halite, syn (WL: 1.5406A_0_). 21-1307: Al OOH Boehmite, syn (WL: 1.5406A_0_). 22-2211: Al (OH)_3_ Bayerite, syn (WL: 1.5406A_0_). 33-0018: Al (OH)_3_ Gibbsite, syn (WL: 1.5406A_0_). X-ray diffraction analysis was also showed in Supplementary Material.

X-ray diffractograms of four aluminum oxyhydroxide samples without terminal resterilization and with terminal resterilization for 30, 60, and 120 min are shown in Figure 1. The broad X-ray diffraction peak indicates that all four aluminum oxyhydroxide samples had poor crystallinity. The angle of X-ray diffraction for the plane indices at 20 is characteristic of Boehmites, i.e., aluminum oxyhydroxide (Al(OH)_3_).

The results obtained from the X-ray diffraction analysis for the plane indices at 20 are summarized in Table 3. First, the angle of X-ray diffraction of aluminum oxyhydroxide without resterilization and with resterilization for a period of 30, 60, and 120 min was 13.412°, 13.495°, 13.625°, and 13.699°, respectively. The angle of X-ray diffraction for the crystalline aluminum oxyhydroxide was 14.485°. Obviously, the angle of X-ray diffraction was gradually shifted to the crystalline aluminum oxyhydroxide when the time of terminal resterilization increased from 30 to 120 min. Second, the lattice spacing (Ǻ) from the angle of X-ray diffraction for aluminum oxyhydroxide without resterilization and with resterilization for 30, 60, and 120 min was 6.5966 Ǻ, 6.5563 Ǻ, 6.4938 Ǻ, and 6.4589 Ǻ, respectively. Clearly, the longer the duration of terminal resterilization, the smaller the lattice spacing. This indicates that the density of aluminum oxyhydroxide may be increased by an additional terminal sterilization process. Third, the degree of full width at half maximum (FWHM) of aluminum oxyhydroxide without resterilization and with resterilization for 30, 60, and 120 min was 4.79°, 4.59°, 4,42°, and 4.21°, respectively. A lower FWHM was measured for aluminum oxyhydroxide with additional sterilization treatment. The degree of FWHM was reduced as the duration of terminal resterilization gradually increased. The lower the FWHM, the less amorphous the aluminum oxyhydroxide. According to the X-ray diffraction data, the structure of aluminum oxyhydroxide in the final container changed after going through the additional sterilization process. Similar results were observed in previous work [16,19,20,21].

### 2.4. The Adsorption Capacity of Aluminum Oxyhydroxide with BSA

The amount of BSA adsorbed to aluminum oxyhydroxide with and without additional terminal sterilization treatment is reported in Table 4. The results clearly indicate that the longer the resterilization treatment, the less BSA is adsorbed by aluminum oxyhydroxide. For aluminum oxyhydroxide without terminal resterilization treatment, a total amount of 1.38 mg BSA was adsorbed by 1 mg of aluminum oxyhydroxide. The amount of adsorption decreased to 1.21, 1.11, and 0.94 mg of BSA for aluminum oxyhydroxide with resterilization for 30, 60, and 120 min, respectively. The corresponding percentages of decrease in adsorption were 2.3%, 19.6%, and 3 1.9%, respectively. Similar results were reported by S.L. Nail et al. and H. Masood et al. [16,17,22].

X-ray diffraction analysis showed that the morphology of aluminum oxyhydroxide after resterilization was gradually changed to a less amorphous structure. Although the zeta potentials of aluminum oxyhydroxide were about the same, the morphology of the aluminum oxyhydroxide structure has changed, modifying its capacity for BSA adsorption. The more amorphous the aluminum oxyhydroxide, the more accessible surface area for BSA adsorption.

### 2.5. The Langmuir Adsorption Isotherm of Aluminum Oxyhydroxide

**Figure 2:**
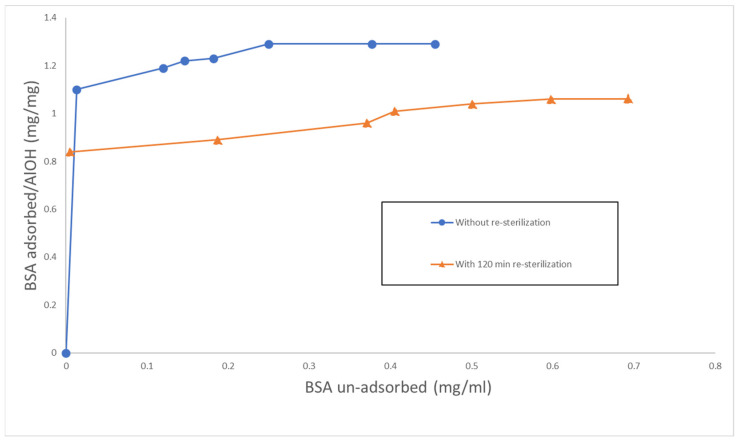
Langmuir isotherm for adsorption of BSA by aluminum oxyhydroxide and without resterilization.

**Figure 3:**
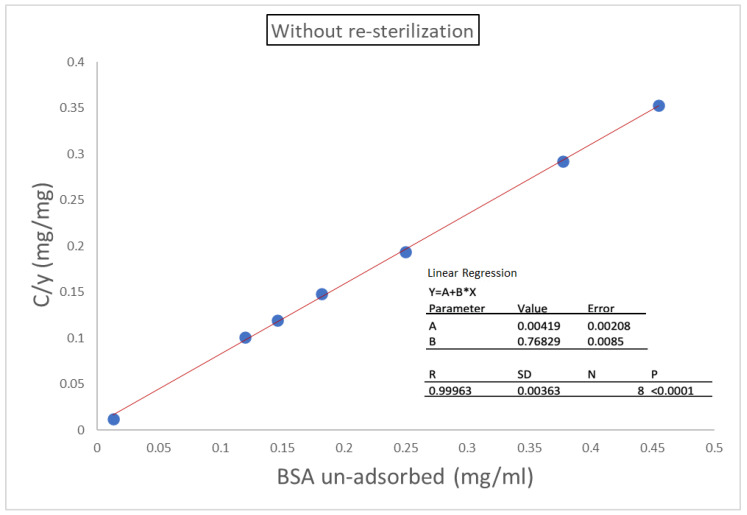

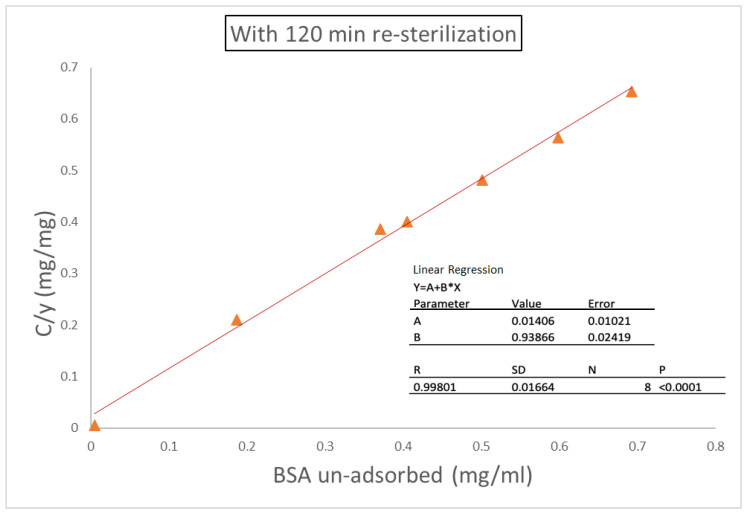
Langmuir isotherm for adsorption of BSA by aluminum oxyhydroxide with and without resterilization.

Figure 2 shows a typical monomolecular adsorption of BSA by aluminum oxyhydroxide, which is frequently referred as the Langmuir type. Obviously, there were differences in the Langmuir adsorption isotherm between aluminum oxyhydroxide with and without terminal resterilization (Figure 2). Figure 3 shows that the adsorption of BSA by aluminum oxyhydroxide with and without resterilization fit well with the Langmuir equation. The correlation coefficient for aluminum oxyhydroxide without resterilization and with 120 min resterilization was 0.999 and 0.998, respectively. The adsorption capacity (y_m_) is the reciprocal of the slope (Figure 3). Therefore, the adsorption capacity was 1.32 mg BSA for aluminum oxyhydroxide without resterilization and 1.10 mg BSA for aluminum oxyhydroxide with 120 min resterilization (Table 5 and Table 6). Furthermore, the corresponding affinity constant (*b*) was calculated as 0.171 and 0.043, respectively. The results show that the adsorption capacity and the affinity of aluminum oxyhydroxide to BSA were reduced by the additional sterilization treatment.

## 3. Materials and Methods

### 3.1. Materials

A concentrated aluminum oxyhydroxide bulk at 15 mg/mL of pharmaceutical grade was used and previously sterilized at 121 °C for 30 min. Bovine serum albumin (BSA) with a molecular weight of approximately 69,000 was purchased from Sigma Chemicals Company (St. Louis, MO, USA). A bicinchoninic acid (BCA) protein assay kit was purchased from Pierce Part No. 23225.

### 3.2. Methods

#### 3.2.1. Sample Preparation and Resterilization

Concentrated aluminum oxyhydroxide was aseptically diluted with saline solution and filled into a 3 mL borosilicate container. A sample with the concentration of aluminum oxyhydroxide of 2.0 mg/mL was used in this study. Aluminum oxyhydroxide samples were resterilized at 121 °C for 30, 60, or 120 min by Finn-Aqua 61215. After resterilization, all aluminum oxyhydroxide samples were stored at 2–8 °C for further study.

#### 3.2.2. Zeta Potential Measurement

Zeta potential measurements were performed on a Coulter DELSA 440SX (Coulter Electronics, Hialeah, FL, USA). The instrument was calibrated according to the Coulter standard before each experiment. The resolution parameter was set at a frequency 20 Hz of peak distances from four different scatter-light angles: 8.6°, 17.1°, 25.6°, and 34.2°. The mobility and zeta potential (mV) were computed based the Doppler electrophoretic light scattering when the particle moved under an electrical field. The mean values from each angle were calculated and compared. Three of each aluminum oxyhydroxide sample were used directly from the 3 mL glass container without dilution for zeta potential measurement.

#### 3.2.3. pH Measurement

The pH of aluminum oxyhydroxide suspension in a 3 mL glass container with and without resterilization was measured by a Corning pH meter (model 125). Daily pH measurements were conducted over a period of one week and compared.

#### 3.2.4. Adsorption of Bovine Serum Albumin with Aluminum Oxyhydroxide 

Bovine serum albumin (BSA) was prepared at a concentration of 6.7 mg/mL, and the pH was adjusted to 6.0 ± 0.1 using 0.02 N HCl. The concentration of BSA was measured by BCA assay.

An HP Chemstation 8453 spectrophotometer was used for the BCA assay.

Triplicate samples of BSA and aluminum oxyhydroxide were mixed in a one-to-one ratio to a total volume of 1.0 mL. Then, sa mples were incubated at room temperature for one hour. Subsequently, samples were transferred to a Spin-X tube and centrifuged at a speed of 4000 rpm for 15 min (Beckmen GS-6R). After centrifugation, the unadsorbed BSA in the supernatant was collected for die concentration determination. In the meantime, samples without aluminum oxyhydroxide were also prepared and analyzed. The amount of BSA adsorbed to aluminum oxyhydroxide can be determined as follows:

Adsorbed BSA to aluminum oxyhydroxide (mg/mg aluminum oxyhydroxide) = (Total BSA (mg) − Un-adsorbed BSA (mg))/Total aluminum oxyhydroxide (mg).

Five consecutive adsorption experiments were conducted. The mean adsorption concentration was calculated in mg BSA/mg aluminum oxyhydroxide.

#### 3.2.5. X-Ray Powder Diffraction Study

An X-ray powder diffraction study was conducted by an outside company (Accurel Systems International Corp., Sunnyvale, CA, USA). Aluminum oxyhydroxide samples were prepared for X-ray diffraction analysis by transferring the precipitated aluminum oxyhydroxide from the ten vials to a clean background-free silica wafer. Then, aluminum oxyhydroxide samples were dried in a desiccator by vacuum.

X-ray diffraction measurements were carried out on a Siemens D500 diffractometer (Siemens D500) and operated at 44 kV × 37 mA. A Cu-Kα (λ = l.54056 Ǻ) radiation source and LiF monochrornator with a soller slit system were used. Diffractograms were obtained on random powder mounts from 5 to 65°. The angle of X-ray diffraction (2θ), lattice spacing (Ǻ), and full width at half maximum (FWHM) of resterilized and non-resterilized aluminum oxyhydroxide samples were compared.

#### 3.2.6. Adsorption Isotherm Study

Concentrations of BSA ranging from 0.8 to 1.6 mg/mL were prepared and mixed with aluminum oxyhydroxide in final containers for the adsorption isotherm study. Unresterilized aluminum oxyhydroxide samples and samples sterilized for 120 min were used and compared. The adsorption capacity of aluminum oxyhydroxide and its affinity constant were calculated based on the Langmuir adsorption isotherm and compared using the Langmuir adsorption equation:

*c*/*y* = 1/*by_m_*+ *c*/*y_m_*

where:

*c* = the concentration of BSA in the solution at adsorption equilibrium (mg/mL);

*y* = the amount of BSA (*x*) in milligrams adsorbed by one milligram (*m*) of aluminum oxyhydroxide (i.e., *y* = *x*/*m*);

*Y_m_* = adsorption capacity; and

*b* = affinity constant.

## 4. Conclusions

Herein, we demonstrated that terminal resterilization results in differences in aluminum oxyhydroxide in the final container using methods including pH measurement, adsorption of BSA, Langmuir adsorption isotherm, and X-ray diffraction analysis.

The physicochemical properties of aluminum oxyhydroxide in the final container showed measurable changes after an additional sterilization treatment. Following an additional sterilization, aluminum oxyhydroxide gradually changed to a less amorphous structure. These structural changes are associated with changes in protein adsorption capacity, affinity for BSA, and the pH of the aluminum oxyhydroxide solution, causing the crystallization and likely biopersistence in the macrophage, muscle, and the brain.

## Figures and Tables

**Table 1:** The zeta potential of aluminum oxyhydroxide with and without terminal resterilization treatment.

Aluminum Oxyhydroxide	Without Resterilization	30 min Resterilization	60 min Resterilization	120 min Resterilization
Zeta Potential (mV)	41	39	42	41

**Table 2:** The pH measurement of aluminum oxyhydroxide solutions with and without resterilization treatment.

Aluminum Oxyhydroxide	Day 1	Day 2	Day 3	Day 4	Day 5	Day 6	Day 7	Mean
Without Resterilization	6.1	6.1	6.2	6.2	6.2	6.1	6.2	6.2
30 min Resterilization	5.9	5.8	5.9	5.9	5.9	5.8	5.9	5.9
60 min Resterilization	5.8	5.8	5.8	5.9	5.8	5.8	5.8	5.8
120 min Resterilization	5.8	5.7	5.8	5.8	5.8	5.8	5.7	5.8

**Table 3:** X-ray diffraction analysis data of aluminum oxyhydroxide with and without resterilization treatment.

Aluminum Oxyhydroxide	Position, AlOOH (020)				FWHM	
	2θ (°)	Δx	d (Ǻ)	Δx	2θ (°)	Δx
Without Resterilization	13.412		6.5966		4.79	
30 min Resterilization	13.495	0.083	6.5563	−0.0403	4.59	−0.2
60 min Resterilization	13.652	0.243	6.4938	−0.1028	4.42	−0.37
120 min Resterilization	13.699	0.287	6.4589	−0.1377	4.21	−0.58

Δx: (Resterilization—without resterilization).

**Table 4:** The adsorption of BSA by aluminum oxyhydroxide with and without resterilization treatment.

Aluminum Oxyhydroxide	Essay 1 mg BSA/mg AlOH	Essay 2 mg BSA/mg AlOH	Essay 3 mg BSA/mg AlOH	Essay 4 mg BSA/mg AlOH	Essay 5 mg BSA/mg AlOH	Essay 6 mg BSA/mg AlOH	Mean mg BSA/mg AlOH
Without Resterilization	1.34	1.38	1.38	1.36	1.42	1.39	1.38
30 min Resterilization	1.19	1.13	1.31	1.17	1.26	1.20	1.21
60 min Resterilization	1.09	1.11	1.15	1.12	1.09	1.14	1.12
120 min Resterilization	0.91	0.96	0.95	0.95	0.93	0.91	0.94

**Table 5:** The Langmuir adsorption of BSA by aluminum oxyhydroxide with and without resterilization treatment.

**Aluminum Oxyhydroxide without Resterilization**			
**BSA (mg/mL)**	**BSA-Unadsorbed (mg/mL) (*c*)**	**BSA Adsorbed/AlOH (mg/mg) (*y*)**	***c*/*y***
1.0	0.013	1.10	0.01181
1.1	0.120	1.19	0.10084
1.2	0.146	1.22	0.11967
1.3	0.182	1.23	0.14796
1.4	0.250	1.29	0.19379
1.5	0.377	1.29	0.29224
1.6	0.455	1.29	0.35271
**Aluminum Oxyhydroxide with Resterilization at 121 °C for 120 min**			
**BSA (mg/mL)**	**BSA Unadsorbed (mg/mL) (*c*)**	**BSA Adsorbed/AlOH (mg/mg) (*y*)**	***c*/*y***
1.0	0.005	0.84	0.00595
1.1	0.187	0.89	0.21011
1.2	0.371	0.96	0.38646
1.3	0.405	1.01	0.40099
1.4	0.501	1.04	0.48173
1.5	0.598	1.06	0.56415
1.6	0.693	1.06	0.65377

**Table 6:** The Langmuir adsorption of BSA by aluminum oxyhydroxide with and without resterilization treatment.

Aluminum Oxyhydroxide	Adsorption Capacity BSA/AlOH (mg/mg)	Affinity Constant (μg/mL)^−1^
Without resterilization	1.32	0.171
With resterilization at 121 °C for 120 min	1.10	0.043

## Supplementary Materials

The following are available online at https://www.mdpi.com/article/10.3390/molecules28020584/s1, X-ray diffraction analysis of aluminum oxyhydroxide (2θ scale) with (a) and without resterilization treatment (b–d) for 30, 60, and 120 min, respectively. 5-0628: NaCl Halite, syn (WL: 1.5406A0). 21-1307: Al OOH Boehmite, syn (WL: 1.5406A0). 22-2211: Al(OH)_3_ Bayerite, syn (WL: 1.5406A0). 33-0018: Al(OH)_3_ Gibbsite, syn (WL: 1.5406A0).

## Sample Availability

Samples of the compounds are not available from the authors.
