# Supplementary figures and images for: Effect of Autoclaving on the Physicochemical Properties and Biological Activity of Aluminum Oxyhydroxide Used as an Adjuvant in Vaccines
Source: Molecules. 2023 Jan 6;28(2):584. doi: 10.3390/molecules28020584 (PMC9862765; doi:10.3390/molecules28020584)

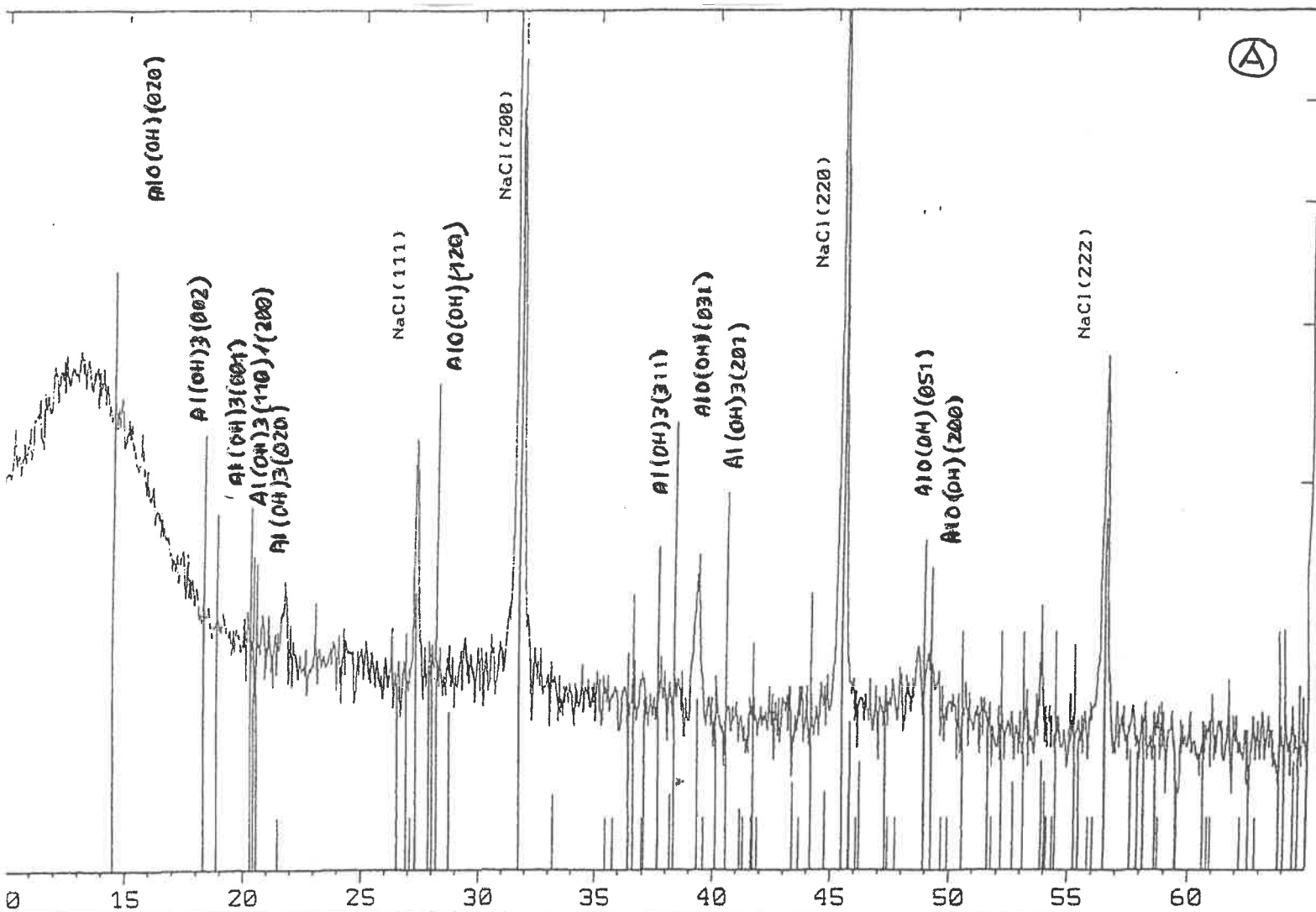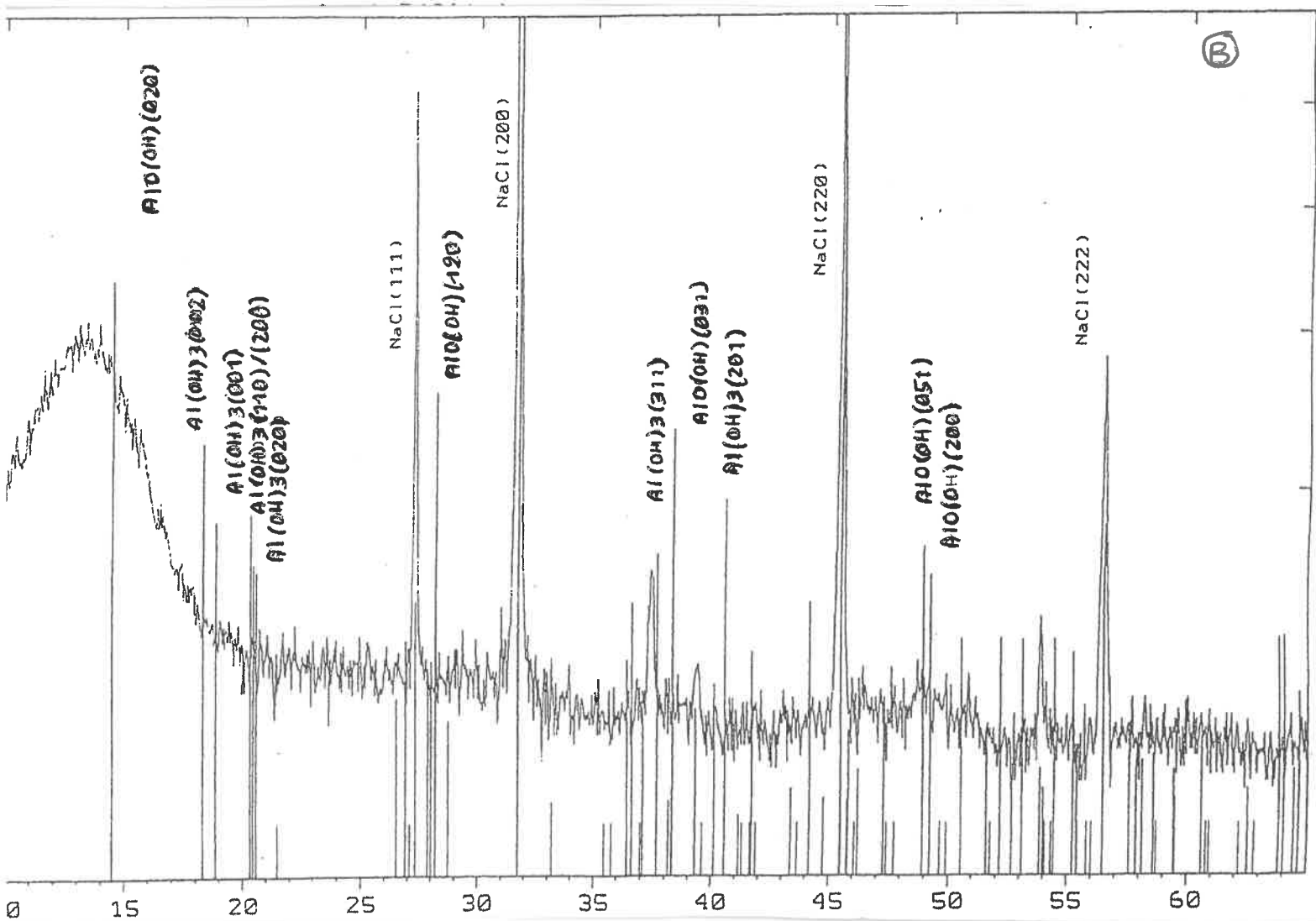

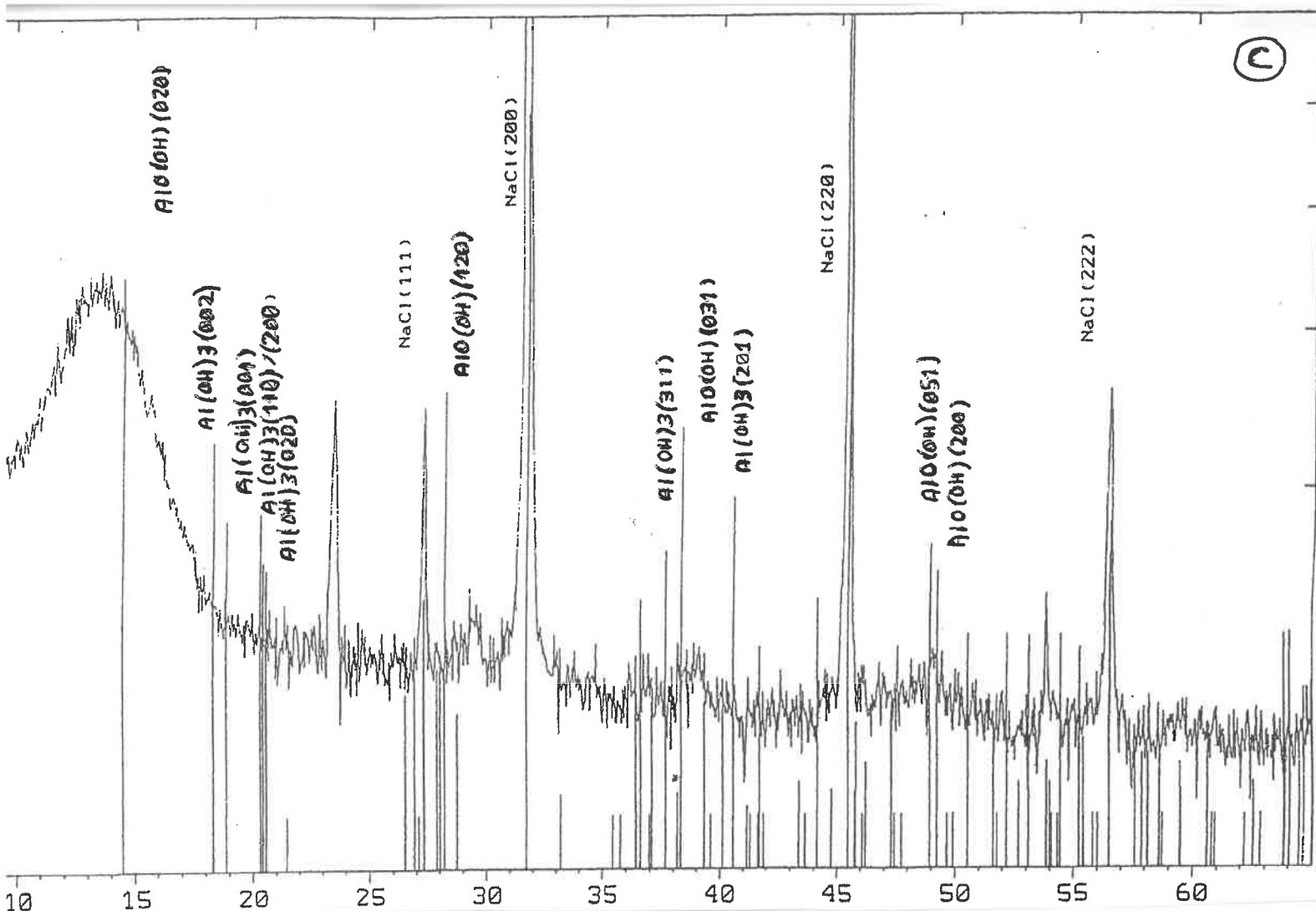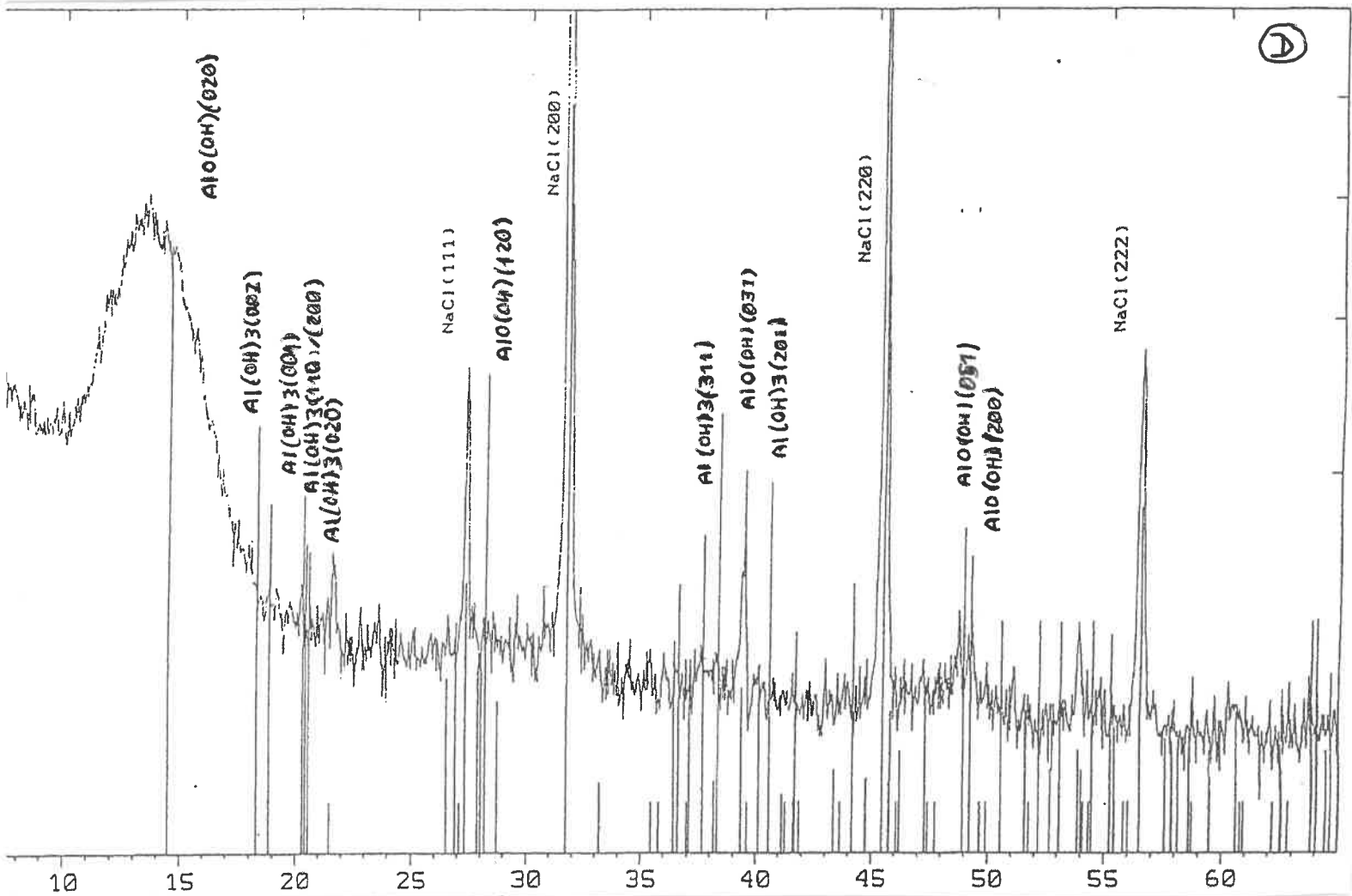

Supplement: Supplementary file 1 [file molecules-28-00584-s001.zip › molecules-2125157-supplementary.pdf]
